# Stress behaviour and physiology of developing Arctic barnacle goslings (*Branta leucopsis*) is affected by legacy trace contaminants

**DOI:** 10.1098/rspb.2018.1866

**Published:** 2018-12-12

**Authors:** Isabella B. R. Scheiber, Brigitte M. Weiß, Margje E. de Jong, Anna Braun, Nico W. van den Brink, Maarten J. J. E. Loonen, Eva Millesi, Jan Komdeur

**Affiliations:** 1 Behavioural and Physiological Ecology, Groningen Institute for Evolutionary Life Sciences, University of Groningen, 9747 AG Groningen, The Netherlands; 2 Department of Behavioural Biology, Faculty of Life Sciences, University of Vienna, 1090 Vienna, Austria; 3 Behavioural Ecology Research Group, University of Leipzig, 04103 Leipzig, Germany; 4 Department of Primatology, Max Planck Institute for Evolutionary Anthropology, 04103 Leipzig, Germany; 5 Arctic Centre, University of Groningen, 9718 CW Groningen, The Netherlands; 6 Department of Toxicology, Wageningen University, 6700 EA Wageningen, The Netherlands

**Keywords:** legacy trace metal contamination, stress coping, acute stress behaviour, HPA corticosterone metabolites, Arctic, barnacle goose (*Branta leucopsis*)

## Abstract

Natural populations are persistently exposed to environmental pollution, which may adversely impact animal physiology and behaviour and even compromise survival. Responding appropriately to any stressor ultimately might tip the scales for survival, as mistimed behaviour and inadequate physiological responses may be detrimental. Yet effects of legacy contamination on immediate physiological and behavioural stress coping abilities during acute stress are virtually unknown. Here, we assessed these effects in barnacle goslings (*Branta leucopsis*) at a historical coal mine site in the Arctic. For three weeks we led human-imprinted goslings, collected from nests in unpolluted areas, to feed in an abandoned coal mining area, where they were exposed to trace metals. As control we led their siblings to feed on clean grounds. After submitting both groups to three well-established stress tests (group isolation, individual isolation, on-back restraint), control goslings behaved calmer and excreted lower levels of corticosterone metabolites. Thus, legacy contamination may decisively change stress physiology and behaviour in long-lived vertebrates exposed at a young age.

## 1. Introduction

One concern that challenges the health and well-being of natural populations is that they are persistently exposed to environmental pollution of anthropogenic sources [1,2]. It is becoming increasingly clear that this affects animal physiology and behaviour in a range of contexts (reviewed in [3]) and may ultimately result in reduced reproductive success and population declines [4–7]. Particularly polar regions, affected most strongly by global climate change [8,9], are fragile and vulnerable to ecological degradation, as they are less capable of self-regeneration and recovery due to their overall low temperatures [10].

An appropriate response to acute stress calls into action physiological and behavioural changes to maximize immediate survival [11]. The short-term activation of the hypothalamic–pituitary–adrenal (HPA) axis leads to a rise in glucocorticoids (i.e. cortisol, corticosterone) and facilitates adaptive physiological and behavioural reactions. Long-term activation, however, results in chronically elevated glucocorticoid levels, which may impair launching of the stress response in case of an acute challenge [12]. Chronic stress may then affect the immune system, behaviour and reproductive performance [13], and even cause death (reviewed in [14]).

Pollutants can target various parts of the endocrine system (e.g. [15]), including the HPA stress axis ([7,14] for reviews). Elevated trace metal exposure may occur near active and abandoned mines due to emission and spreading of mine tailings [16] or calamities [17]. Free-ranging white storks (*Ciconia ciconia*), exposed to an industrial accident known as the Doñana disaster (Aznalcóllar, Spain), showed higher levels of corticosterone during handling and restraint than did birds from a reference site [18], with maximum levels of corticosterone being positively related to lead (Pb) [18]. Likewise, exposure to mercury (Hg) has been related to changes in glucocorticoid production [19], although in some studies corticosterone seemed to increase [20], decrease [21,22] or remain unchanged [23,24] (for a recent review see [25]). Adult male, but not female, common loons (*Gavia immer*), for example, showed a positive relationship between (Hg) levels and circulating corticosterone levels during handling and restraint, whereas other steroid hormones (testosterone, oestradiol) remained unaffected [19]. To some extent this variation might be related to the time of exposure, as individuals exposed continuously and from an early age may be particularly affected [21].

Effects of contaminants on physiological systems may translate into behavioural changes and reduced fitness. For instance, disruption of prolactin secretion as a result of (Hg) exposure lowered reproductive success in black-legged kittiwakes (*Rissa tridactyla*) through reduced paternal care [26]. Likewise, nesting near a long-term exposed (Hg) contaminated river correlated with smaller clutches and lower fledging success in female blue birds (*Sialia sialis*), possibly due to lower provisioning rates of males [27]. To the best of our knowledge, however, no studies measured effects of trace metal pollution on behaviour in response to acute stressors, neither in the wild nor in the laboratory. In particular, behaviours such as vigilance (e.g. looking up), escape (e.g. movement, pecking) and, in social species, behaviours facilitating group cohesion (e.g. vocalizations, re-establishing spatial proximity) are likely to be beneficial in the short term, but might cause a negative energy budget or call the attention of a potential predator when performed excessively [28].

The available literature provides important indications for an effect of trace metal pollution on physiology and behaviour, but as stated above, the link between pollution, physiology and behaviour in response to acute stressors is currently lacking. Earlier studies either experimentally added various amounts of (Hg) to the food of captive individuals or were non-experimental field studies that could not control whether observed effects were due to pollutants or potential differences in individual quality. Therefore, experimental field studies considering potential differences in individual quality are needed to understand the effects of environmental pollution on stress physiology and behaviour in natural environments. Hence, here we experimentally quantified how exposure to pollutants from a historic coal mine affected stress-related behaviours and excreted immuno-reactive corticosterone metabolites (CORTm) in developing barnacle goslings raised in their natural Arctic environment by human foster parents.

Coal mining began on the High Arctic Svalbard archipelago in the early 1900s and two mines are still in operation. One prominent coal mine implosion, ‘the Kings Bay Affair’, occurred in 1962 near Ny-Ålesund and resulted in immediate termination of mining in 1963 [29]. Coal was remediated only close to the village, while the mine area itself was left alone. (Hg) is present in measurable amounts in soil and vegetation at relatively low levels compared to other Arctic sites, but still significantly different between the former mining area and previously not exposed areas. Furthermore, we showed in a complementary study that feeding in the former mining area resulted in higher levels of (Hg) in liver and concentration-related variations in D2-receptors in the brains of barnacle goslings [30].

For this study we submitted barnacle goslings raised either on polluted or clean grounds to three experimental stress tests: a group isolation, an open field individual isolation, and a back test, at an age of 13 to 23 days. We quantified stress-related behaviours during the tests and collected dropping samples for determination of immuno-reactive corticosterone metabolites (CORTm) prior to and after tests to (i) determine baseline levels over development as well as (ii) an acute stress response immediately after the tests. We predicted exposed goslings to show (1) stress-related behaviours during stress tests to a higher extent than their control siblings, (2) disrupted group cohesion during the group test as a result of increased stress-related behaviours and (3) an elevated absolute CORTm baseline over time as well as a stronger increase in their adrenocortical response after the tests than controls.

## 2. Methods

### (a) Study population

We studied barnacle geese from a breeding population in Kongsfjord, Svalbard, as described in detail in [31]. For this study we used the same goslings as in [30] which were human-raised in two groups (*n* = 8 per group). For this purpose, we removed two goslings per nest (‘sibling groups’) during hatching on the contamination-free island Indre Breøyane (Svalbard Archipelago, 79°00′ N, 12°06′ E [31]), approximately 9 km offshore from the village of Ny-Ålesund (78°55′ N, 11°56′ E) and marked them individually with coloured leg bands. From each pair of siblings, we randomly sorted one into the exposed group, the other one into the control group. This renders initial differences in physiology or pollution levels between experimental groups highly unlikely. Sex was determined genetically from blood samples after termination of the experiment. The exposed group consisted of five males and three females, the control group of four males and four females, respectively. Until they were 4 days old, we kept all 16 goslings as a large group which was allowed to feed in meadows in and in close proximity to the village. Once they were 5 days old, the exposed group was led daily for a minimum of 5 h to feed in a trace metal exposed mine area, sporting large coal heaps, 1.5 km to the southeast of the village, while the control group was raised in the opposite direction (1.9 km northwest) on clean locations around Ny-Ålesund ([31] for details). Both groups of goslings were allowed to graze freely during the walk and at the final destination. Since the desertion of the mine, typical Svalbard tundra vegetation (*Carex* spp., *Saxifraga* spp., mosses) regrew and now also wild geese use this area for feeding ([30] for details). Besides their separated daily walks in the assigned groups, all 16 goslings were kept together in one big group for the rest of the day. To avoid potential differences in parental effects of human foster parents between groups, goslings were accompanied by four humans (A.B., M.E.d.J., N.W.v.d.B., I.B.R.S.) in a round-robin fashion on a daily basis. The foster parents ensured the well-being of the animals by checking that goslings fed properly and had access to water, and by providing shelter and predator protection throughout daytime (i.e. continuously from 06.00 to 23.00). Overnight, goslings were housed inside a predator-safe pen with an infrared lamp and were provided with a commercial diet for young waterfowl (Anseres I food, starter pellet, Kasper Faunafood, Woerden, The Netherlands) *ad libitum*, a supply of fresh vegetation from the clean area, and water ([31] for details). Goslings were checked daily for health and well-being, and we found no differences in various immune parameters between the groups ([31] for details). Furthermore, their body mass was taken on a daily basis. In order to minimize handling, goslings were trained to voluntarily step on a digital balance. Goslings of the two groups did not differ in their growth rates (electronic supplementary material, results, figure S1).

At the end of the experiment (i.e. when goslings were 23 days old) they were sacrificed through decapitation [30,31]. On account of this, single goslings were removed from the group and carried to a laboratory by one of their human foster parents in order to reduced stress levels before they were sacrificed. Immediately after decapitation, goslings were dissected to collect liver and brain tissue samples for determination of mercury and neuro-receptor levels, respectively [30].

### (b) Behaviour during stress tests

When goslings were 13, 18 and 23 days old, goslings entered three well-established stress tests (see below). All tests were video recorded and, in all but one case (individual behaviour during the group isolation), analysed by two observers naive to the group background of the individuals, but familiar with goose behaviour.

Both the control and exposed group received a *group isolation* first (i.e. when 13 days old), in which one group was left in a novel fenced area of 2 × 2 × 1 m (length × width × height) with a heat lamp as well as food and water *ad libitum* for one hour. From the video recordings we scored behaviours indicative of stress *per individual*: (a) number of ‘look ups’ as a measure for vigilance, (b) movement patterns, generally shown as stereotyped pacing in the confined area, and (c) the number of pecks against the fence per 4-min interval. We further quantified on a *group-level* group density and group cohesion (i.e. the number of subgroups) every minute (see electronic supplementary material methods for details).

Furthermore, we performed an *open field individual isolation*, where we separated one individual gosling at a time for 20 min in a wooden box (length × width: 0.50 × 0.76 m). To control for habituation and age effects, one half of the sibling groups received the test when they were 18 days old, the other half when they were 23 days old. From video recordings, we again measured (a) number of jumps, (b) number of look ups, (c) ‘border crosses’, (d) number of pecks against the box and (e) the number of distress calls [32] in 4 min intervals (see electronic supplementary material methods for details).

Finally, we placed goslings in an ‘*on-back*’ position (‘*back test*’) and measured the time until the gosling righted itself. Again, one half of the sibling groups received the back test when they were 18 days old, the other half when they were 23 days old (see electronic supplementary material methods for details).

### (c) Immuno-reactive corticosterone metabolites (CORTm)

When goslings where 3, 9, 12, 17 and 22 days old, we collected a minimum of three dropping samples [33] of all individuals in their respective feeding areas over a 3 h period to determine baseline corticosterone metabolites. To determine the acute physiological stress response, we collected droppings for 3 h [33] immediately after the stress tests (see electronic supplementary material methods for details). All droppings were frozen within 1 h, and later on analysed using an enzyme immuno assay (EIA, see electronic supplementary material methods for details). We computed the average change (Δ CORTm) between baselines collected one day before a stress test and after the respective stress test as CORTm (Ø_samples stress test_) − CORTm (Ø_samples_ _baseline_) per individual per test.

### (d) Statistical analyses

We computed generalized linear mixed models (GLMMs) and linear mixed models (LMMs) in R version 3.2.3 [34]. To investigate the impact of the raising condition on *individual behaviour* we fitted separate models for each stress-related behaviour per 4 min time bin per individual as response variables. For individual behaviours during the group isolation we fitted the raising condition (exposed versus control) and its interaction with time (i.e. the time bin) as fixed effects test predictors, and time and sex as fixed effects control predictors. To investigate effects of the raising condition on *group behaviour*, we fitted group density and the number of subgroups (as a proxy for group cohesion) as response variables with raising condition and its interaction with time as fixed effects predictors. For behaviours during the individual isolation we fitted the raising condition and its interaction with time as fixed effects test predictors and further included the interaction of raising condition with age as test predictor. Age, time and sex were fitted as fixed effects control predictors. In all models we assessed if autocorrelation was an issue and, if necessary, included an autocorrelation term as an additional fixed effects control (see electronic supplementary material methods for details).

We analysed the impact of raising condition on stress hormones by fitting mean individual baseline CORTm and Δ CORTm after the stress tests as response variables and raising condition and its two-way interactions with age and test type (group isolation, individual isolation, back test) as fixed effects test predictors. We further fitted age, test type and sex as fixed effects control predictors and, in the model on Δ CORTm, the corresponding baseline CORTm value as an additional fixed effects control predictor (see electronic supplementary material methods for details on model formulation as well as computation of *p*-values and checks of model assumptions).

Finally, we conducted a Student's paired *t*-test to investigate if exposed and control siblings differed in the time they need to right themselves during the physical restraint in a forced ‘*on-back*’ position by using the paired *t*-test online calculator (http://www.sthda.com/english/rsthda/paired-t-test.php). All tests were conducted two-tailed.

## 3. Results

### (a) Effects of exposure on behaviour in various stress tests

#### (i) Group isolation

**Figure 1.**
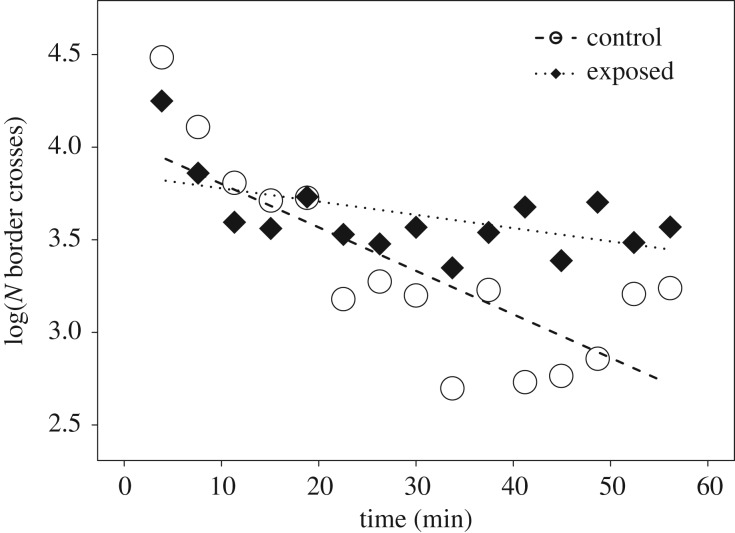
Number of border crosses (log-transformed) of exposed (black diamonds) and control (white circles) goslings over the 60 min group isolation. Points depict the raw values binned into 4 min intervals, lines show the fitted model conditional on all other predictors being at their average (dotted: exposed goslings, *n* = 8; dashed: control goslings, *n* = 8).

Raising condition (exposed versus control) influenced *individual* stress related-behaviours during the 1 h group isolation. The two groups differed in individual levels of vigilance, i.e. the number of look-ups (likelihood ratio test (LRT): *χ*^2^ = 7.865, d.f. = 2, *p* = 0.02). Exposed goslings looked up more often than control goslings (median: exposed 59.5, control 34 times per hour; table 1). Furthermore, movement patterns differed between the two groups in a time-dependent manner (LRT: *χ*^2^ = 30.513, d.f. = 2, *p* < 0.001; table 1). As time progressed, all goslings moved around less, but this decrease in movements was far less pronounced in exposed than in control goslings (figure 1). There was no difference between the groups, however, in the number of stereo-typed pecks against the enclosure's fence throughout the whole duration of the test (median: exposed 79.5, control 63 times per hour; LRT: *χ*^2^ = 0, d.f. = 2, *p* = 1).

**Table 1.** Effects of exposure to contaminants on individual and group behaviour. Results were obtained with GLMMs, test statistics were derived from likelihood ratio tests. Reference levels for factorial predictors were ‘control’ (condition), ‘18 days’ (age) and ‘female’ (sex). Estimates depict effects of levels in parentheses relative to reference levels. Interactions are denoted by an asterisk. Non-significant interactions were removed from the models, values of non-significant interactions represent results of terms before removal from the model. All other results stem from models excluding non-significant interactions. estim. = model estimate, CI lo = CI 2.5%, CI hi = CI 97.5%, a.c.term = autocorrelation term, exp. = exposed. Significant terms are marked in bold, trends in italics. d.f. for all tests results is 1.

		group isolation						individual isolation					
model	predictor	estim.	s.e.	CI lo	CI hi	*χ*^2^	*p*	estim.	s.e.	CI lo	CI hi	*χ*^2^	*p*
*jumps*	intercept							−1.42	0.93	−4.38	0.44	^a^	^a^
	condition (exp.)							**1**.**18**	**0**.**18**	**0**.**84**	**1**.**80**	**12**.**48**	**<0.001**
	age (23 d)							**2**.**65**	**1**.**23**	**0**.**06**	**6**.**29**	**3**.**98**	**0**.**046**
	time^b^							−0.02	0.15	−0.36	0.31	0.02	0.882
	sex (male)							**−1**.**06**	**0**.**22**	**−1**.**95**	**−0**.**65**	**11**.**38**	**0**.**001**
	condition*age							−0.17	0.50	−1.19	0.89	0.12	0.734
	condition*time							−0.39	0.28	−0.98	0.22	1.76	0.184
*look ups*	intercept	0.89	0.19	0.54	1.26	^a^	^a^	54.35	10.91	30.77	78.87	^a^	^a^
	condition (exp.)	**0**.**58**	**0**.**18**	**0**.**25**	**0**.**74**	**7.43**	**0.006**	**19**.**01**	**6**.**55**	**4**.**54**	**33**.**76**	**5**.**81**	**0**.**016**
	age (23 d)							6.32	13.95	−24.96	37.25	0.20	0.653
	time^b^	0.04	0.06	−0.08	0.17	0.45	0.504	−**6**.**35**	**1**.**84**	**−10**.**41**	**−2**.**30**	**7**.**37**	**0**.**007**
	sex (male)	−0.21	0.15	−0.53	0.13	1.61	0.204	−2.09	7.83	−27.96	14.54	0.07	0.79
	condition*age							11.01	12.52	−16.69	39.53	0.74	0.39
	condition*time	−0.08	0.12	−0.33	0.18	0.44	0.507	4.45	3.37	−2.82	11.72	1.58	0.209
*border crosses*	intercept	3.59	0.10	3.79	3.82	^a^	^a^	3.49	0.31	2.82	4.15	^a^	^a^
	condition (exp.)	0.33	0.21	−0.11	0.79	^a^	^a^	*0*.*48*	*0*.*23*	−*0*.*02*	*1*.*00*	*3*.*54*	*0*.*06*
	age (23 d)							−0.51	0.38	−1.35	0.33	1.64	0.201
	time^b^	−0.41	0.03	−0.47	−0.34	^a^	^a^	−*0*.*28*	*0*.*13*	−*0*.*58*	*0*.*00*	*3*.*78*	*0*.*052*
	sex (male)	−0.49	0.13	−0.78	0.39	1.37	0.242	−0.21	0.27	−0.82	0.35	0.61	0.435
	a.c.term	0.12	0.02	0.07	0.16	25.53	<0.001						
	condition*age							0.47	0.43	−0.48	1.45	1.11	0.292
	condition*time	**0**.**28**	**0**.**05**	**0**.**19**	**0**.**37**	**28.08**	**<0.001**	0.22	0.14	−0.09	0.57	2.12	0.145
*group area*	intercept	9.05	0.13	8.79	9.31	^a^	^a^						
	condition (exp.)	−1.17	0.19	−1.53	−0.80	^a^	^a^						
	time^c^	−0.02	0.00	−0.03	−0.01	^a^	^a^						
	a.c.term	0.21	0.05	0.12	0.31	18.62	<0.001						
	condition*time	**0**.**03**	**0**.**01**	**0**.**01**	**0**.**04**	**20.19**	**<0.001**						
*n sub-groups*	intercept	0.88	0.17	0.54	1.20	^a^	^a^						
	condition (exp.)	−0.68	0.27	−1.22	−0.16	^a^	^a^						
	time^c^	−0.01	0.01	−0.02	0.00	^a^	^a^						
	a.c.term	0.06	0.06	−0.06	0.18	0.9	0.343						
	condition*time	**0**.**02**	**0**.**01**	**0**.**00**	**0**.**03**	**4.37**	**0.037**						

^a^values not presented because of having a very limited interpretation for intercepts and terms comprised in significant interactions.

^b^*z*-transformed, the original values were 8.00 ± 4.33 (mean ± s.d.) for the group isolation and 2.50 ± 1.13 (mean ± s.d.) for the individual isolation.

^c^*z*-transformed, the original values were 30.00 ± 17.68 (mean ± s.d.).

**Figure 2.**
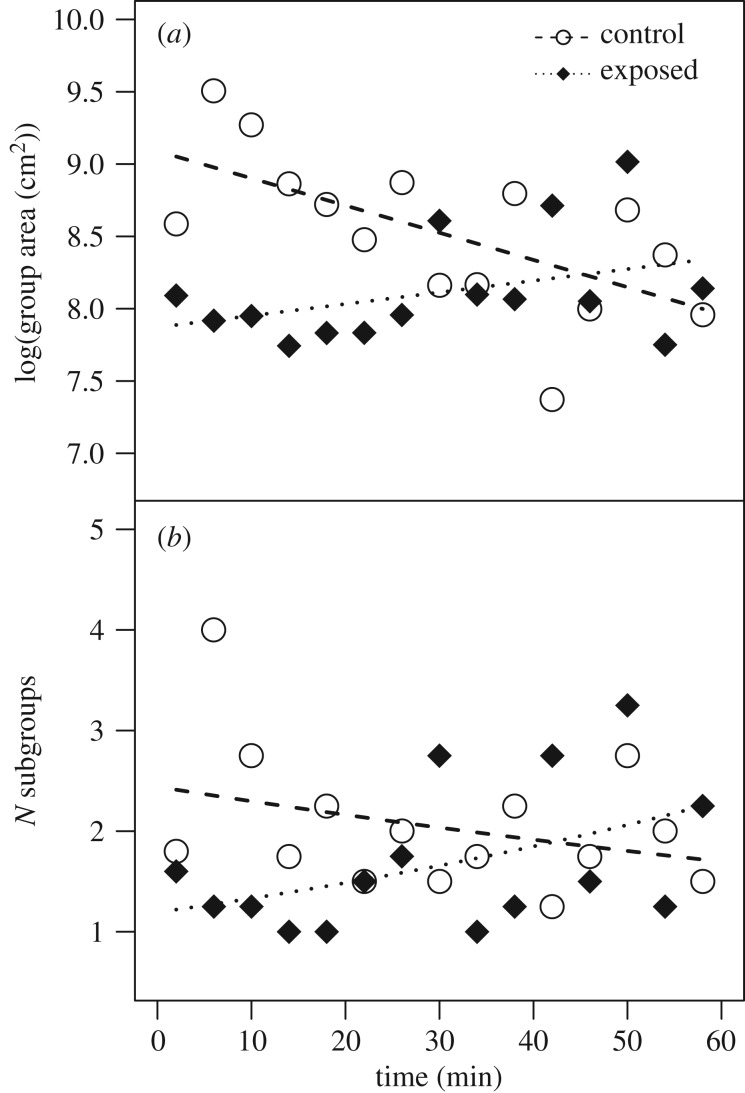
Group density and cohesion of exposed (black diamonds) and control (white circles) goslings over the course of the group isolation. Group density (*a*) is expressed as the area (in cm^2^, log-transformed) taken up by the group, i.e. low values indicate high density and vice versa. Group cohesion (*b*) is expressed as number of subgroups, i.e. low values indicate high cohesion and vice versa. Points depict the raw values binned into 4 min intervals, lines show the fitted model conditional on all other predictors being at their average (dotted: exposed goslings, *n* = 8; dashed: control goslings, *n* = 8).

The raising condition also significantly affected group density (LRT: *χ*^2^ = 35.465, d.f. = 2, *p* < 0.001), whereby this effect interacted with time (table 1). In particular, the group area decreased in the control group as the isolation progressed (i.e. goslings gradually moved closer together). In the exposed group, on the other hand, the group area marginally increased over time (figure 2*a*). Group cohesion also differed between the exposed and control groups in a time-dependent manner (LRT: *χ*^2^ = 6.522, d.f. = 2, *p* = 0.038; table 1). At the start of the experiment the exposed group consisted of fewer subgroups, i.e. was more cohesive, but split into more subgroups as time progressed. The opposite was true for the control group: here the number of subgroups decreased marginally (figure 2*b*). This subgrouping pattern remained similar but became non-significant when excluding a potential outlier (electronic supplementary material, results, figure S2).

#### (ii) Individual isolation

Similar to the group isolation, stress-related behaviours differed between the exposed and control group when the goslings were isolated individually in a cage for 20 min. They differed significantly in the number of attempts to jump out of the cage (LRT: *χ*^2^ = 14.316, d.f. = 3, *p* = 0.003). Exposed goslings attempted to escape more often than did the controls irrespective of the goslings' age or the time progression during the test (median: exposed 18 times, control 1.5 times; table 1). Yet, overall, 23-day-old goslings jumped more often than 18 day old goslings and males jumped less than females, regardless of raising condition (median: 18 days 0 times, 23 days 28 times; males 2 times, females 15 times; table 1).

Vigilance also differed between raising conditions during the individual isolation (LRT: *χ*^2^ = 8.125, d.f. = 3, *p* = 0.044). Exposed goslings looked up significantly more often than did controls irrespective of the goslings’ age or the time progression during the test (median: exposed 291.5 times, control 192 times; table 1). Regardless of the raising condition both groups looked up less as time went on (table 1). Furthermore, movement patterns of goslings during the individual isolation tended to differ between the groups (LRT: *χ*^2^ = 6.778, d.f. = 3, *p* = 0.079). Although both groups generally tended to move less over time, exposed goslings tended to move around more than did controls (median: exposed 122.5 times, control 119.5 times; table 1). There was no interaction between raising condition and the age of the goslings or the time progression in the test (table 1). Raising condition neither had effects on the number of stereo-typed pecks against the cage (median: exposed 11 times, control 23 times; LRT: *χ*^2^ = 2.162, d.f. = 3, *p* = 0.54) nor the number of distress calls (median: exposed 438 times, control 471 times; LRT: *χ*^2^ = 3.293, d.f. = 3, *p* = 0.349).

#### (iii) Back test

Goslings of the exposed and control group did not differ in the time they needed to turn over after being physically constrained in the forced ‘*on-back*’ position (mean ± s.e.: exposed: 9.6 s ± 1.1, control 12.1 s ± 3.5; paired *t*-test: *t* = 0.717, d.f. = 7, *p* = 0.497).

### (b) Effects of exposure on baseline and stress-induced corticosterone metabolites (CORTm)

**Figure 3.**
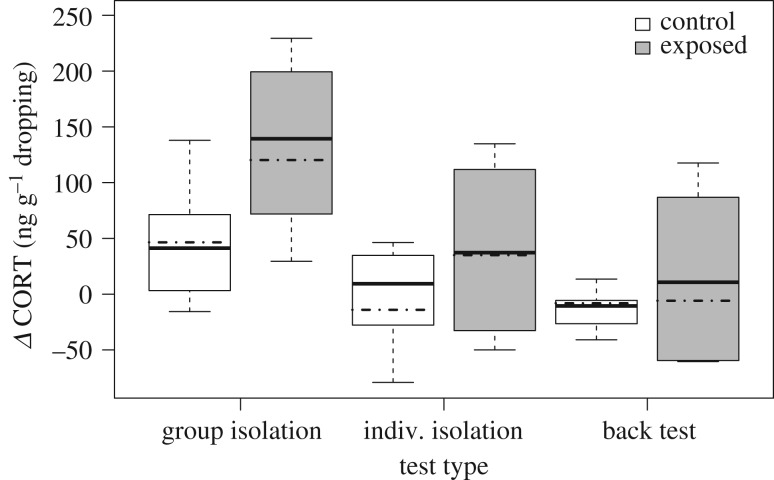
Δ CORTm (ng CORTm/g dropping) of exposed (grey bars, *n* = 8) and control (white bars, *n* = 8) goslings after three stress tests (group isolation, individual isolation, back test). Positive values indicate an increase in CORTm compared to baseline levels, while negative values indicate a decrease. Boxplots show medians and first and third quartiles. Lower (upper) whiskers are located at the larger (smaller) value of the minimum (maximum) × value or the first (third) quartile ± 1.5 × interquartile range. Dash-dotted lines show the fitted model conditional on the average age of the respective test type and all other predictors being at their average.

Whereas raising condition had no effect on baseline CORTm throughout ontogeny (LRT: *χ*^2^ = 2.542, d.f. = 2, *p* = 0.281), it significantly contributed to the observed variation in Δ CORTm after the stress tests (LRT: *χ*^2^ = 18.033, d.f. = 4, *p* = 0.001). The effect of raising condition was modulated by test type (table 2, figure 3), with exposed goslings showing a stronger increase in CORTm after the group isolation (LRT: *χ*^2^ = 4.475, d.f. = 1, *p* = 0.034) and individual isolation (LRT: *χ*^2^ = 14.536, d.f. = 1, *p* < 0.001) but not after the back test (LRT: *χ*^2^ = 0.135, d.f. = 1, *p* = 0.714). Irrespective of the raising condition, Δ CORTm was higher in older goslings and tended to be lower in males than in females (table 2).

**Table 2.** Effects of exposure to contaminants on the rise in excreted corticosterone metabolites (Δ CORTm) after stress tests. Results were obtained with an LMM, test statistics were derived from likelihood ratio tests. Reference levels for factorial predictors were ‘control’ (condition), ‘group isolation’ (test type) and ‘female’ (sex). Estimates depict effects of levels in parentheses relative to these reference levels. Non-significant interactions were removed from the models, values of non-significant interactions represent results of terms before removal from the model. All other results stem from models excluding the non-significant interaction. CI low = CI 2.5%, CI high = CI 97.5%. Significant terms are marked in bold, trends in italics. d.f. for all test results is 1.

predictor	estimate	s.e.	CI low	CI high	*χ*^2^	*p*
intercept	17.486	0.794	15.88	19.16	^a^	^a^
condition (exposed)	2.283	0.746	0.73	3.95	^a^	^a^
test type					^a^	^a^
(indiv. isolation)	−5.068	0.996	−7.19	−3.03		
(back test)	−4.060	1.090	−6.40	−1.80		
age^b^	**1**.**936**	**0**.**478**	**0**.**94**	**2**.**96**	**10.816**	**0.001**
*sex (male)*	*−0*.*793*	*0*.*394*	*−1*.*63*	*0*.*06*	*3.38*	*0.066*
baseline CORTm	**−0**.**061**	**0**.**006**	**−0**.**06**	**−0**.**05**	**27.039**	**<0.001**
condition*test type					**6.339**	**0.042**
(exposed:indiv. isolation)	0.171	1.086	0.10	0.51		
(exposed:back test)	−2.099	1.037	−2.10	−1.16		
condition*age	1.705	1.189	−1.20	4.50	1.33	0.249

^a^values not presented because of having a very limited interpretation.

^b^*z*-transformed, the original values were 18.00 ± 4.13 (mean ± s.d.).

## 4. Discussion

In this experimental field study, we show that feeding at a site polluted decades ago affects behavioural and physiological responses to acute stressors in developing barnacle goslings. This is remarkable because the contamination levels in the abandoned mine area are on the lower end relative to other, more recently polluted, sites in the Arctic [30]. Furthermore, exposure time of contamination to goslings was extremely short, only spanning a total of 19 days. Yet this is a potentially sensitive time window of development in this long-lived species, which is supported by effects that mining exposure had on neuroreceptor levels in these goslings [30].

### (a) Effects of exposure on stress-related behaviours

All results combined reveal that control goslings were behaviourally either less stressed from the start and/or were able to calm down as time in the tests progressed, indicating more efficient acute stress coping compared to exposed individuals. Control goslings responded with a reduction of stress-related behaviours over the course of the individual and group isolation, particularly in attempts to escape, vigilance and movements.

The acute stress response is flexible, and does not only depend on the type of stressor, but more importantly on how an individual perceives it [35]. Launching an appropriate stress response following an acute stressor is adaptive and crucial for health and survival [35], but chronical elevation of glucocorticoids imbalances the momentary beneficial components of HPA activation and results in a state where individuals no longer respond appropriately to life-threatening stimuli (reviewed in [35]). The elevated numbers of stress-related behaviours might be advantageous in the short term, for example, when being more vigilant results in fleeing faster from a potential predator, but detrimental in the long term, if higher responses of exposed goslings repeatedly lead to dispensable, energetically costly, actions. Unnecessary escape attempts of single young make them easier prey, as parents can no longer protect them, and reduce time spent feeding, as those behaviours cannot be performed concurrently. After being exposed to the major bioavailable form of (Hg) methylmercury (MeHg), zebra finches reacted more strongly to a perceived threat of predation and risked starvation, as exposed birds began to feed later, resulting in lower body masses [28]. Here, an inspection of body mass data collected over development provided no indications for different body mass gain in the two groups (electronic supplementary material, results, figure S1), probably because both groups received supplemental food when outside their respective grazing areas. This could have allowed exposed goslings to replenish potential food shortages resulting from either inefficient feeding or an inability to use nutrients aptly.

Intriguingly, we also found differences in the area the groups used and the number of subgroups formed, a potential proxy for group density and cohesion, respectively. In social species, one effective mechanism of stress reduction is social support, where the presence of social allies reduces stress [36]. Not only during the individual isolation, but more importantly also during the group isolation, exposed goslings moved around more than control goslings. This did not appear to disrupt group cohesion: at the beginning of the group isolation we found fewer subgroups in the exposed group, although this difference became non-significant after excluding a potential outlier in the control group. Yet, the erratic movements displayed by the exposed goslings over the course of the test could potentially cause social disruption when a stressor continues, as they eventually split into more subgroups. Particularly relevant for precocial species, such as geese [37], movements away from the family in the wild cause a higher predation risk and demand more energy already in very young individuals, reinforcing the inappropriate stress behaviours under continuous stress described above. Such inappropriate stress responses during early development may be a mechanistic explanation for effects of early-life exposure to contaminants on reproduction as observed in white storks [38] (but see [39]).

### (b) Effects of exposure on CORTm

This study provides further support that contamination modulates endocrine systems, specifically functioning of the HPA axis, because we found substantial variation in Δ CORTm after the stress tests among exposed versus control goslings. This is particularly relevant, as the range of change in the stress response, and not the exposure to stressors *per se*, ultimately determines fitness [38]. On average, all control goslings responded in a predicted manner by showing relatively stable Δ CORTm levels over all three tests, with only slight variations in Δ CORTm between individuals. In contrast, the values of exposed individuals were significantly higher in the group and individual isolation. Δ CORTm levels did not differ between exposed and control goslings in the back test, presumably a mild stressor, where we also found no differences in behaviour. In all three tests, however, variation of Δ CORTm levels in exposed individuals was much larger relative to control goslings. Notably, the range of responses in exposed goslings did not only comprise an upregulation of CORTm but in some instances an actual downregulation, which is indicative of a more erratic, and potentially dysfunctional, stress response [7,14,35].

Chick age may play an important role as mercury accumulates with age [40], although growth dilution may actually result in (temporary) lower concentrations in developing chicks [30]. As already low concentrations of pollutants can impair the HPA system, the duration of exposure and/or nestling age are important in determining long-term negative effects [18,19,22,41]. In mercury contaminated areas, for example, baseline suppression occurred in adrenal corticosterone in juvenile tree swallows (*Tachycineta bicolor*) [21,22] at the end of the nestling period. The authors suggested that those effects are most evident once the endocrine systems are fully developed, as effects were more prominent in older nestlings [21].

We also found that age of goslings influenced Δ CORTm patterns, but it did so irrespective of the raising condition. In fact, Δ CORTm values in both groups were highest after the group isolation, when all goslings were youngest (i.e. 13 days old) and exposure time was shortest. Yet this may result from the group isolation being the first test for all goslings. Goslings therefore may have perceived this test as the strongest stressor overall. In the later tests, where goslings were tested individually and the age at the tests was randomized, goslings of both groups responded more when older (i.e. 23 versus 18 days old). A detailed analysis of how test type, order and age interact with raising condition to affect the HPA system warrants further studies.

Whereas stress CORTm differed between the two groups, this variation was absent in baseline CORTm, which illustrates that exposure does not necessarily lead to more stressed animals overall but rather to altered responses under stressful situations. This corroborates findings in a companion study involving the same goslings, where baseline plasma corticosterone levels (representing a single point in time rather than an integrated measure over time) did not differ between exposed and control goslings, but levels increased in both groups after the goslings were individually isolated [31]. Hence, both studies strengthen the fact that baseline glucocorticoid level were not (yet) altered by the past contamination. In the arctic summer, barnacle goslings were shown to excrete CORTm in a phase-shifted diel pattern, which might be indicative of a pre-maturely developed HPA system subject to change in older goslings [42]. It is possible that a suppressive effect of (Hg) on baseline CORTm levels might only become evident once the HPA system shows the characteristic adult corticosterone secretion pattern.

Generally, studying trace metal contamination is confounded by the fact that they tend to occur combined, either with other metals and/or with organic pollutants [43]. In the present study we cannot ascertain which metals and/or pollutants are responsible for our findings, but van den Brink *et al.* [30] found increased levels of Hg in droppings (mean ± s.e.: exposed 0.08 ± 0.02, control 0.048 ± 0.01 mg kg^−1^ dry weight [30]) and hepatic Hg (mean ± s.e.: exposed 0.030 ± 0.003 versus 0.022 ± 0.002 mg kg^−1^ dry weight [30]) in the same exposed goslings, which further correlated positively with D2-neuroreceptors in the brain [30]. Blocking D2 receptors in the brain is known to reduce stress-related behaviours [44] and to affect the contribution of those receptors to HPA functioning [45].

By combining behavioural and physiological approaches, this study adds important insights into effects of environmental contamination on stress coping abilities in a highly vulnerable ecosystem. Our study shows that past contamination persists in the long term at levels sufficient to elicit behavioural and physiological responses to acute stressors in developing animals already after a few days of exposure. Investigating these consequences is necessary to fill yet another gap in our understanding of the impacts of trace metals on threats to birds, wildlife in general and humans.

## Supplementary Material

ESM Methods detailed

## Supplementary Material

ESM Results detailed

## Supplementary Material

Complete data set

## Data accessibility

All relevant data are provided in the paper and its supporting information files (see electronic supplementary material, data table).
