# Supplementary material for: Stress behaviour and physiology of developing Arctic barnacle goslings (Branta leucopsis) is affected by legacy trace contaminants
Source: Proc Biol Sci. 2018 Dec 12;285(1893):20181866. doi: 10.1098/rspb.2018.1866 (PMC6304058; doi:10.1098/rspb.2018.1866)
Supplement: ESM Methods detailed [file rspb20181866supp1.docx]

**ESM Methods:**

**Behaviour During Stress tests**

Group Isolation

The first test we performed was a *group isolation*, where one group was left in a novel fenced area of 2 x 2 x 1 m (length x width x height) with a heat lamp as well as food and water *ad libitum* for one hour. From the video recordings we first continuously quantified various behaviours indicative of stress *per individual*: a) number of ‘look ups’ as a measure for vigilance, and b) movement patterns, generally shown as stereotyped pacing in the confined area. To assess movement patterns, we subdivided the cage into 16 0.5 x 0.5 m squares (length x width) by overlaying a grid onto the computer screen when analysing the video recordings later on to count the number of crosses from one square into another. A cross was completed, once the individual gosling stepped over a respective border with both legs. This ensured that the body’s centre of gravity was in the new, adjacent square. We further quantified c) the number of pecks against the fence. To be able to take into account potential changes over time, we scored all occurrences of the respective behaviours per 4-minute interval, *i.e.* for a total of 15 intervals per individual over the one-hour isolation. We had planned to also score positive and negative social interactions, *i.e.* greetings and agonistic pecks, between the goslings, but these behaviours only occurred rarely (control group: n_greetings_ = 9, n_pecks_ = 0; exposed group: n_greetings_ = 5, n_pecks_ = 16), therefore we refrained from analysing these any further.

In a further analysis of the video-recordings of the group isolation, we quantified on a *group-level* group density and group cohesion. Group density was measured as the area that the eight goslings took up in the test compartment by determining the smallest ellipse that could be placed around all goslings. The length and width of the ellipse were estimated to the nearest 5 cm by placing a grid over the video image that subdivided the cage into 0.25 x 0.25 m sections. We calculated the area of the ellipse as length/2*width/2*π. Group cohesion was measured as the number of subgroups that formed, whereby individuals within one body length of one another were scored as belonging to the same subgroup. We scored group density and cohesion every minute throughout the entire group isolation, resulting in 61 data points each over the one-hour isolation.

Open field individual isolation

Furthermore, we performed an *open field individual isolation*, where we separated one individual gosling at a time for 20 min in a wooden box (length x width: 0.50 x 0.76 m). We divided the box in four equal quadrants (length x width: 0.125 x 0.19 m). To control for habituation and age effects, one half of the sibling groups (colour rings: orange, red, green, black), drawn at random, received the individual isolation when they were 18 days old, the other half when they were 23 days old. From video recordings, we again measured stress behaviors: (a) number of jumps b) number of look ups, (c) ‘border crosses’ (d) number of pecks against the box and (e) the number of distress calls, *i.e.* vocalizations of goslings uttered in case of need [1]. Due to technical problems with video recordings during some trials, however, only the first 16 min of the test were analysed later on. As for the group isolation, behaviours were scored in 4-min intervals, *i.e.* for a total of 4 intervals per individual over the course of the individual isolation.

Back test

Finally, we performed a physical restraint in a forced ‘*on-back*’ position (‘*back test’*). One half of the sibling groups (colour rings: white, yellow, blue, brown) received the back test when they were 18 days old, the other half when they were 23 days old. Two of us (AB, IBRS) separated an individual gosling from the group and one of us (AB) turned the gosling on its back and held it on the ground until it ceased struggling. One the count of three, AB removed her hands, and IBRS set a timer. As soon as the gosling righted itself and walked back towards the group, the timer was stopped. The ‘on-back’ restraint is considered a stressor, because a gosling’s escape from a potential predator is inhibited when turned on its backside.

**Baseline and stress induced immuno-reactive corticosterone metabolites (CORTm)**

In regular intervals, *i.e.* when goslings where 3, 9, 12, 17 and 22 days old, we collected a minimum of three dropping samples [2] of all individuals in their respective feeding areas over a three-hour period to determine baseline corticosterone metabolites (B CORTm1 – B CORTm5) of goslings over time. Baselines B CORTm3 – B CORTm5 were collected one day prior to the stress tests and served as controls for stress-induced values to determine the acute physiological stress response (S CORTm1 – S CORTm3). The latter was determined from droppings collected for three hours [2] immediately after the stress tests, based on the gut passage time of barnacle geese [3]. All droppings were frozen within 1 hour, and later-on shipped frozen to the Dept. of Behavioural Biology, University of Vienna (Austria) for analyses. Immuno-reactive corticosterone metabolites, CORTm, were quantified using an enzyme immuno assay (EIA, for detailed description of the methodology and validation of the EIA for barnacle geese, see [3]). As variation between individuals as well as individual samples tends to be high in geese [2], we computed the average change (Δ CORTm) per individual between the droppings collected matched in time one day before (control) and up to three hours after the respective stress test. This was calculated as CORTm (∅_samples stress test_) − CORTm (∅_samples_ _baseline_) per individual per test. Δ CORTm values can either be positive, indicating a higher corticosterone metabolite excretion after the stress test, equal zero, or be negative, indicating a higher corticosterone metabolite excretion during baseline collection performed one day prior to the test [2, 4].

**Statistical Analyses**

We computed Generalized Linear Mixed Models (GLMMs) and Linear Mixed Models (LMMs ) in R version 3.2.3 [5] using the package 'lme4' version 1.1.-11 [6]. To investigate the impact of the raising condition on *individual behaviour during the* *group isolation* we fitted models for ' number of look ups', 'number of border crosses' and 'number of pecks' per 4-minute time bin per individual as response variables (n per model = 16 individuals x 15 time bins = 240). To accommodate the different data distributions and meet model assumptions, 'look ups' were fitted with a Poisson, 'border crosses' (log(+10) transformed) with a Gaussian and 'pecks' (zero-inflated) with a negative binomial error structure. For all three responses we fitted the raising condition (exposed vs. control) and its interaction with time (*i.e.* the time bin) as fixed effects test predictors, and time and sex as fixed effects control predictors.

To investigate effects of the raising condition on *group behaviour*, we fitted the log-transformed area taken up by the goslings every minute (as a proxy for group density) with a Gaussian error structure and the number of subgroups (as a proxy for group cohesion) with a Poisson error structure (n per model = 2 groups x 61 minutes = 122). In both models the raising condition and its interaction with time were fitted as the only fixed effects predictors. As data on group cohesion contained one potential outlier (7 subgroups in minute 8 in the control group) we additionally conducted this analysis without it (see ESM Results, Figure 2).

We conducted analyses of *individual behaviour during the individual isolation* in a similar manner by fitting 'number of jumps', 'number of border crosses' and 'number of pecks' per time bin and individual with a Poisson error structure and 'number of look ups' and 'number of distress calls' (log(+1) transformed) with a Gaussian error structure (n per model = 16 individuals x 4 time bins = 64). As above, we fitted the raising condition and its interaction with time as fixed effects test predictors. As the individual isolation was conducted at different ages of the goslings, we further included the interaction of raising condition with age as test predictor to take into account that differences between exposed and control goslings might only appear later during development (*i.e.* after longer exposure). Age, time and sex were fitted as fixed effects control predictors.

To assess if behavioural data collected closely in time showed temporal autocorrelation (*i.e.* if residuals of data points recorded more closely in time were more similar to each other) we computed autocorrelation terms by first deriving the residuals from the above models. We then averaged residuals for each data point and individual (or group) to all other data points and weighing the contribution of each residual by the time lag to the respective data point. The best fitting standard deviations for the autocorrelation function were obtained by maximizing the likelihood of the model including the autocorrelation term as described in [7]. The resulting autocorrelation term had a positive model estimate, indicative of an actual autocorrelation, in four of the models investigating effects during the group isolation ('border crosses', 'pecks', 'group density', 'group cohesion'). For these four models we thus included the autocorrelation term as an additional fixed effects control.

We analysed the impact of raising condition on stress hormones by fitting mean individual baseline CORTm (log-transformed) at the age of 3, 9, 12, 17 and 22 days and Δ CORTm (square-root(+84) transformed) after three stress tests at the age of 13, 18 and 23 days as Gaussian response variables (baseline: n = 16 individuals x 5 ages = 80; Δ CORTm: n = 16 individuals x 3 ages = 48). In both models we fitted the raising condition and its two-way interactions with age and test type (group isolation, individual isolation, back test) as fixed effects test predictors and age, test type and sex as fixed effects control predictors. In the model on Δ CORTm we fitted the corresponding baseline CORTm value as an additional fixed effects control predictor to account for the dependence of Δ CORTm values on these baselines and any short-term fluctuations therein.

Following [8], we z-transformed all co-variates before running the models. In all but the two models on group density and cohesion, we fitted the identity of the individual and the sibling group as random effects to account for repeated measures, and fitted a maximal random slopes structure to achieve more reliable p-values [9]. We first compared full models comprising all terms with null models lacking the test predictors but containing all other terms using Likelihood ratio tests (LRTs, R function 'anova'). If the full-null model comparison was significant or a trend (p < 0.1), this indicated an effect of the raising condition either by itself or in interaction with other terms. In these cases, we determined the p-values of the fixed effects with LRTs using the R function 'drop 1'. In the case of non-significant interactions, we re-ran the respective model without the interaction(s) to facilitate interpretation of the main terms. In the case of a significant interaction between two factors (*i.e.* raising condition and test type) we further conducted post-hoc tests by computing separate models per test type and assessed the effect of raising condition with LRTs as described above. For all models we checked assumptions of residuals and dispersion, absence of collinearity between predictors (Variance inflation factors using R package 'car' [10]) and model stability. Checks revealed no issues of concern with the exception of moderate overdispersion in the models on look ups during the group isolation (dispersion parameter 2.07) and pecks during the individual isolation (dispersion parameter 1.78). As overdispersion may lead to anti-conservative p-values, we repeated the model on look ups with the observational level as additional random effect to reduce overdispersion. This resulted in a moderately underdispersed model (dispersion parameter 0.42) with highly similar estimates and p-values. The model on pecks during the individual isolation was non-significant and we therefore took no further measures to correct for overdispersion. The model on pecks during the group isolation, on the other hand, was severely underdispersed (dispersion parameter 0.001) and thus has to be considered as very conservative, but did not meet the assumptions of other error distributions.

**References ESM:**

[1] Loth, A., Frigerio, D., Kotrschal, K. & Szipl, G. 2018 Differential responses to gosling distress calls in parental and non-parental greylag geese (*Anser anser*). *J. Ornithol.* **159**, 401-412. (doi:10.1007/s10336-017-1521-0).

[2] Scheiber, I.B.R., Kralj-Fišer, S. & Kotrschal, K. 2005 Sampling effort/frequency necessary to infer individual acute stress responses from fecal analysis in Greylag Geese (*Anser anser*). *Ann. NY Acac. Sci.* **1046**, 154-167. (doi:10.1196/annals.1343.012).

[3] Scheiber, I.B.R., de Jong, M.E., Komdeur, J., Pschernig, E., Loonen, M.J.J.E., Millesi, E. & Weiß, B.M. 2017 Diel pattern of corticosterone metabolites in Arctic barnacle goslings (*Branta leucopsis*) under continuous natural light. *PLOS One* **12**, e0182861. (doi:10.1371/journal.pone.0182861).

[4] Scheiber, I.B.R., Kotrschal, K. & Weiß, B.M. 2009 Benefits of family reunions: Social support in secondary greylag goose families. *Horm. Behav.* **55**, 133-138. (doi:10.1016/yhbeh.2008.09.006).

[5] R Core Team. 2017 R: A language and environment for statistical computing. (Vienna, Austria, R Foundation for Statistical Computing).

[6] Bates, D., Mächler, M., Bolker, B.M. & Walker, S.C. 2015 Fitting linear mixed-effects models using lme4. *J. Stat. Softw.* **67**, 1-48. (doi:10.18637/jss.v067.i01).

[7] Kulik, L., Amici, F., Langos, D. & Widdig, A. 2015 Sex differences in the development of social relationships in rhesus macaques (M*acaca mulatta*). *Int. J. Primatol.* **36**, 353-376. (doi:10.1007/s10764-015-9826-4).

[8] Schielzeth, H. 2010 Simple means to improve the interpretablity of regression coefficients. *Methods. Ecol. Evol.* **1**, 103-113. (doi:10.1111/j.2041-210X.2010.00012.x).

[9] Barr, D.J., Levy, R., Scheepers, C. & Tily, H.J. 2013 Random effects structure for confirmatory hypothesis testing: Keep it maximal. *J. Mem. Lang.* **68**, 255-278. (doi:10.1016/j.jml.2012.11.001).

[10] Fox, J. & Weisberg, S. 2011 *An R Companion to Applied Regression*. 2nd ed. Thousand Oaks, CA, USA, Sage Publications Inc. “Retrieved from http://socserv.socsci.mcmaster.ca/jfox/Books/Companion”
