# Supplementary material for: Stress behaviour and physiology of developing Arctic barnacle goslings (Branta leucopsis) is affected by legacy trace contaminants
Source: Proc Biol Sci. 2018 Dec 12;285(1893):20181866. doi: 10.1098/rspb.2018.1866 (PMC6304058; doi:10.1098/rspb.2018.1866)
Supplement: ESM Results detailed [file rspb20181866supp2.docx]

**ESM Figure 1**


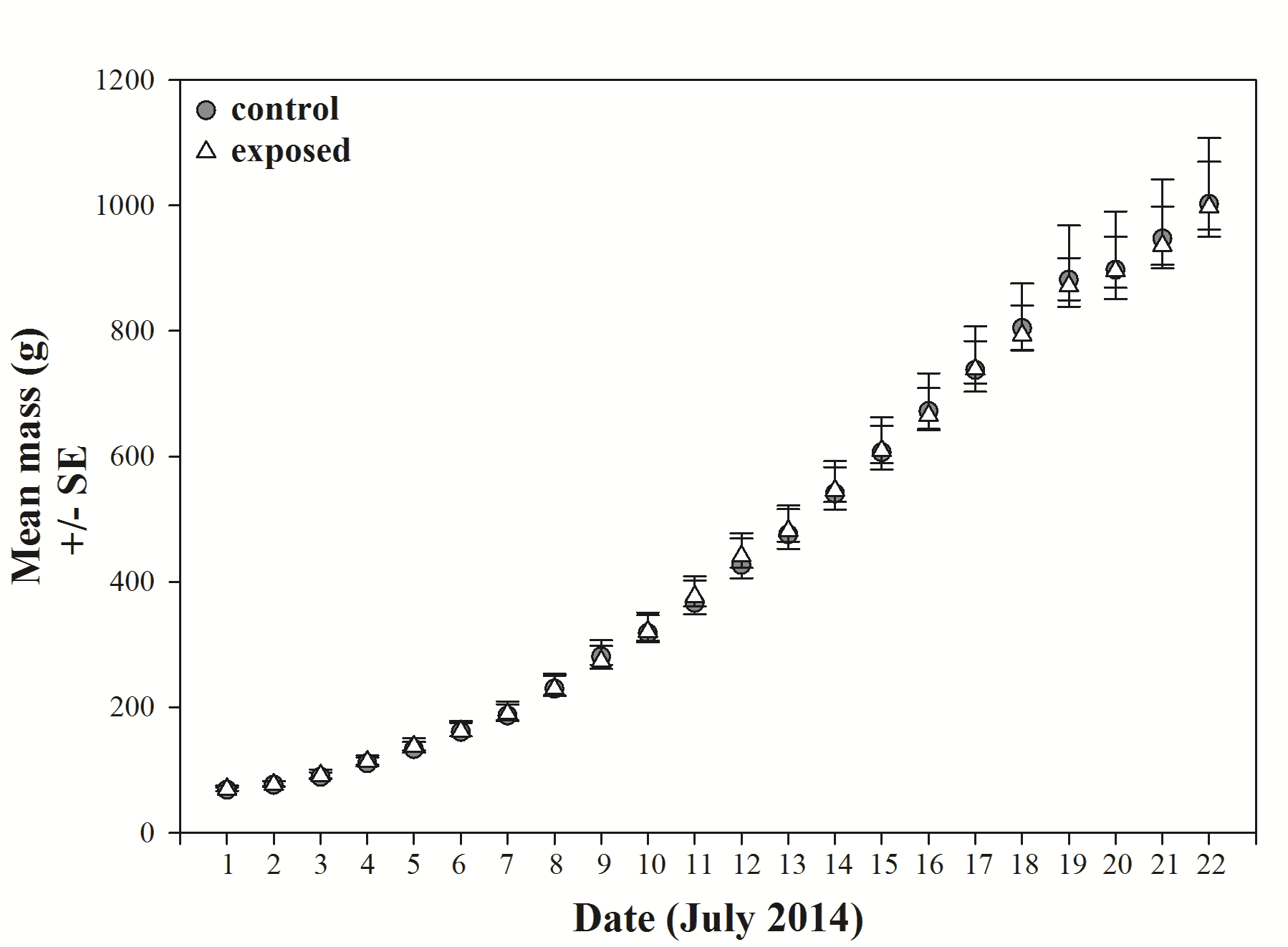


Daily mean mass (g) ± SE of control (grey circle, N = 8) and exposed (white triangle, N = 8) goslings from July 1^st^ to July 22^nd^, 2014.

**ESM Figure 2:**

**Group cohesion during the Group Isolation**


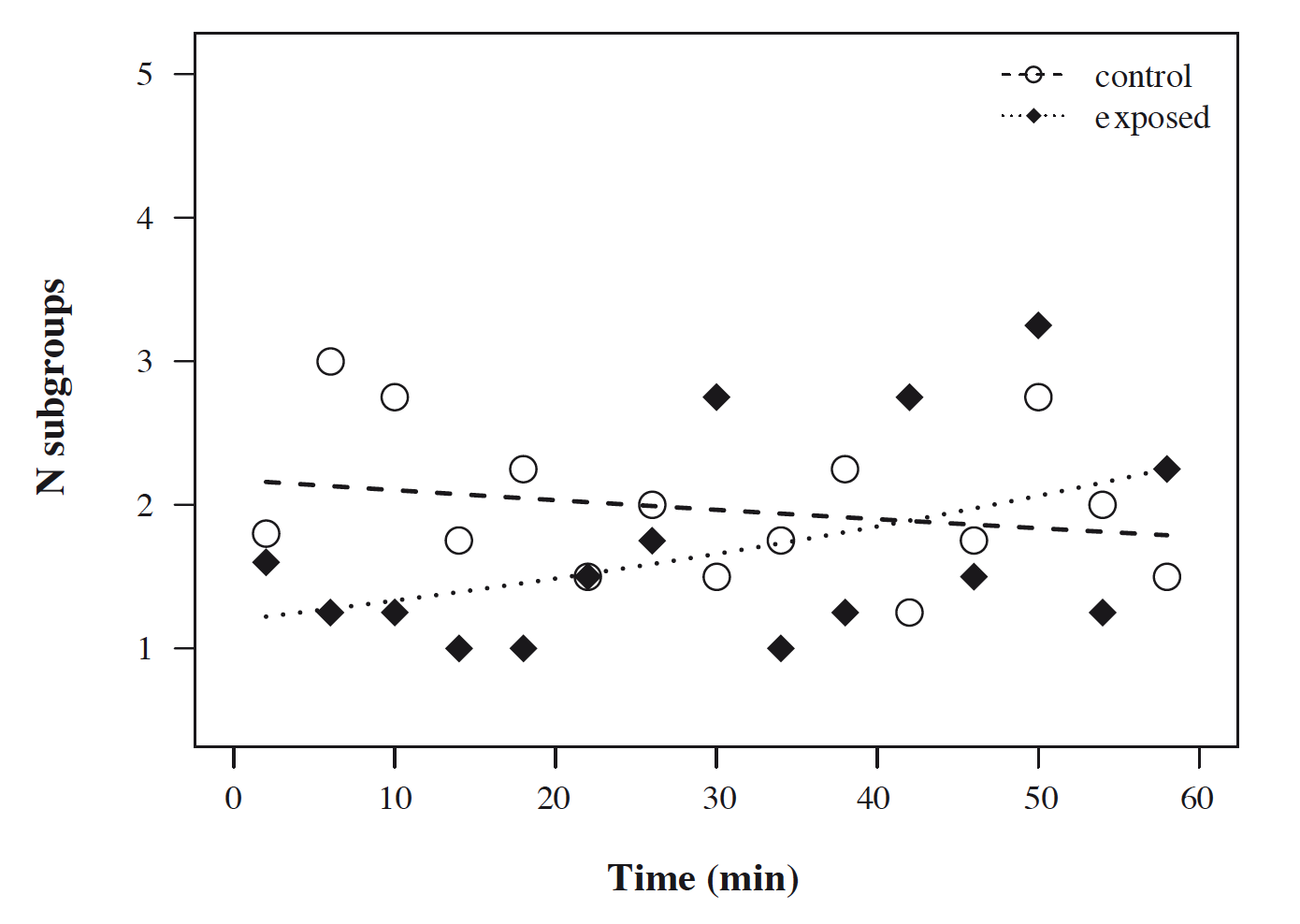
Goslings of both groups split into one to five subgroups over the course of the group isolation, with the exception of one outlier in the control group (7 subgroups in minute 8). When excluding this outlier the general pattern remained similar: the exposed group consisted of fewer subgroups at the start of the experiment, but split into more subgroups as time progressed, while the number of subgroups in the control group marginally decreased over the course of the group isolation (Fig. S1). This effect, however, was no longer significant (interaction condition*time, LRT: χ 2 = 2.99, df = 1, p = 0.084).

Group cohesion of exposed (black diamonds) and control (white circle) goslings over the course of the group isolation, excluding one outlier. Group cohesion is expressed as number of subgroups, *i.e.* low values indicate high cohesion and vice versa. Points depict the raw values binned into 4-minute intervals, lines show the fitted model conditional on all other predictors being at their average (dotted: exposed goslings, N = 8; dashed: control goslings, N = 8).
